# The Number of Metabolic Abnormalities Associated with the Risk of Gallstones in a Non-diabetic Population

**DOI:** 10.1371/journal.pone.0090310

**Published:** 2014-03-05

**Authors:** Chung-Hung Tsai, Jin-Shang Wu, Yin-Fan Chang, Feng-Hwa Lu, Yi-Ching Yang, Chih-Jen Chang

**Affiliations:** 1 Department of Family Medicine, Tainan Municipal An-Nan Hospital-China Medical University, Tainan City, Taiwan; 2 Department of Family Medicine, National Cheng Kung University Hospital, Tainan City, Taiwan; 3 Department of Family Medicine, College of Medicine, National Cheng Kung University, Tainan City, Taiwan; Yonsei University College of Medicine, Republic of Korea

## Abstract

**Aim:**

To evaluate whether metabolic syndrome is associated with gallstones, independent of hepatitis C infection or chronic kidney disease (CKD), in a non-diabetic population.

**Materials and Methods:**

A total of 8,188 Chinese adult participants that underwent a self-motivated health examination were recruited into the final analysis after excluding the subjects who had a history of cholecystectomy, diabetes mellitus, or were currently using antihypertensive or lipid-lowering agents. Gallstones were defined by the presence of strong intraluminal echoes that were gravity-dependent or that attenuated ultrasound transmission.

**Results:**

A total of 447 subjects (5.5%) had gallstones, with 239 (5.1%) men and 208 (6.0%) women. After adjusting for age, gender, obesity, education level, and lifestyle factors, included current smoking, alcohol drinking, regular exercise, hepatitis B, hepatitis C, and CKD, there was a positive association between metabolic syndrome and gallstones. Moreover, as compared to subjects without metabolic abnormalities, subjects with one, two, and three or more suffered from a 35, 40, and 59% higher risk of gallstones, respectively.

**Conclusions:**

Non-diabetic subjects with metabolic syndrome had a higher risk of gallstones independent of hepatitis C or CKD, and a dose-dependent effect of metabolic abnormalities also exists.

## Introduction

Metabolic syndrome is a clustering of cardiometabolic risk factors, such as high blood pressure, hyperglycemia, central obesity, and dyslipidemia. Metabolic syndrome is associated with the development of type 2 diabetes mellitus and cardiovascular disease [1], and also with increased risk of all-cause mortality [2]. It has become one of the major health problems worldwide, with a prevalence of 14–25% [3]–[5].

Gallstones appear to be associated with metabolic syndrome, because type 2 diabetes mellitus, dyslipidemia, and hyperinsulinemia have been reported as well-known risk factors for this condition [6]–[9]. Moreover, gallstones are also a common disorder throughout the world, with a prevalence of 5–30%, varying by region and race [10]–[12]. The formation of gallstones is multifactorial, and in addition to the traditional risk factors, such as female gender, increasing age, obesity, family history of gallstones, ethnicity, oral contraceptive use, number of pregnancies, and menopause [10], hepatitis C infection [13] and chronic kidney disease (CKD) [14] have also been found to be associated with them in recent studies.

Previous studies have shown a positive association between diabetes mellitus and gallstones, including previous-diagnosed and newly-diagnosed diabetes mellitus [9], [15]–[17]. A few reports have examined the association between metabolic syndrome and gallstones in a non-diabetic population [18], [19], although important confounding factors, including hepatitis C infection and CKD, were not considered in these studies. Moreover, the dose-effect of metabolic abnormalities on gallstones had not been studied in non-diabetic subjects. Therefore, the aim of this study was to evaluate whether metabolic syndrome is associated with gallstones, independent of hepatitis C infection or CKD, in non-diabetic population.

## Materials and Methods

The Ethical Committee for Human Research at the National Cheng Kung University Hospital approved the study protocol used in this work (A-ER-101-190). Participants' informed consents were not needed because the data in this research was analyzed anonymously. The institutional review board of the National Cheng Kung University Hospital waived the need for informed consent from the participants because due to using de-linked data for analysis. The study is using a secondary data analysis, and the research data was retrospectively extracted from an existing dataset of self-motivated physical check-up. From January, 2000 to August, 2009, a total of 19,694 participated in the self-motivated health examination in National Cheng Kung University Hospital. The participants received different health check-up package depended on their need, and 3599 subjects who did not receive abdominal ultrasound or complete blood test were excluded. Each participant was asked to complete a standard structured questionnaire, which included demographic data, medical history, medication history, and life style factors included smoking, alcohol drinking, and details of their physical exercise habits, but 5857 participants did not fulfill the complete questionnaires and were excluded. Furthermore, a total of 2050 participants were excluded from this study on the basis of the following criteria: (1) history of diabetes mellitus or newly diagnosed diabetes [fasting plasma sugar (FPG) ≧7.0 mmol/l, 2-hour post load glucose (2-h PG) ≧11.1 mmol/l, or glycated hemoglobulin (HbA1C) ≧6.5%] according to the diagnostic criteria of American Diabetic Association in 2010 [20] (n = 792); (2) less than 20 years old (n = 36); (3) currently using antihypertensive agents (n = 1014); (4) currently using lipid-lowering agents (n = 597); (5) history of cholecystectomy (n = 184); (6) incomplete data (n = 52). A total of 8,188 adults were recruited for the final analysis, which was carried out on the basis of secondary data without personal identification information.

Anthropometric measurements were carried out by experienced nurses. Body weight (to nearest 0.1 kg) and height (to nearest 0.1 cm) were automatically measured using a certified machine (HW-3030, Super-View, Kaohsiung, Taiwan). The body mass index (BMI) was calculated as weight in kilograms divided by the square of height in meters. Waist circumference (WC) was measured from the midway between the lower rib margin and the iliac crest with the subjects standing at the end of normal expiration. Two seated readings of blood pressures were measured with an automatic blood pressure monitor (DINEMAP SX1846, Critikon, California, USA), with the subjects in a supine position after at least 5 minutes of rest.

Smoking habit was classified as current (defined as at least one pack per month, for at least the previous half year) and non-current smoking. Alcohol drinking habit was classified as current (defined as at least once per week, for at least the previous half year) and non-current alcohol drinking. Regular exercise was defined according to the recommendation of the American College of Sport Medicine (ACSM) guideline of vigorous exercise at least three times weekly, intense enough to cause sweating and/or heavy breathing, and/or increase the heart rate to a certain amount [21]. Exercise habit was categorized into two groups: none to less than three times per week, and three times or more per week. Educational level was classified into two groups: less than 12 years, and 12 years or more.

Participants were instructed to fast for at least 10 hours and avoid smoking, alcohol, coffee, and tea on the day of the examination. The laboratory tests included HbA1C, FPG, total cholesterol, triglyceride, high-density lipoprotein cholesterol (HDL-C), aspartate aminotransferase (AST), alanine aminotransferase (ALT), creatinine, urea nitrogen, and albumin. The 75-gm oral glucose tolerance test was performed in a participant without pregnancy or a history of DM, and a 2-h PG sample was obtained. FPG and 2-h PG were determined using the glucose oxidase method (Synchron CX3, Beckman Coulter Inc., California, USA). HbA1C was measured using ion-exchange HPLC (HbA1c, BIO-RAD V-II TURBO Hemoglobin A1c program, Bio-Rad Laboratories, Inc). Total cholesterol, triglyceride,HDL-C, AST, ALT, creatinine, urea nitrogen, and albumin concentrations were measured using the automated analyzer (Roche Modular DP, Roche Diagnostics, Mannheim, Germany). Chronic kidney disease (CKD) was defined as an estimated glomerular filtration rate (eGFR) of less than 60 mL/min per 1.73 m^2^, as calculated by the Modification of Diet in Renal Disease formula [22].
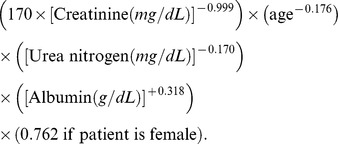



Diagnosis of hepatitis B and C was defined by seropostivity of hepatitis B surface antigen and antibody to hepatitis C virus, which were determined using a chemiluminescent microparticle immunoassay (CMIA) method (Architect i2000SR, Abbott Laboratories, Abbott Park, Illinois, USA).

The diagnosis of gallstones was established on the basis of the results of an abdominal ultrasound examination, which was performed using a convex-type real-time electronic scanner (Xario SSA-660A, Toshiba, Tokyo, Japan) and operated by experienced radiologists who were unaware of the objectives of the study, and blind to the laboratory data. Gallstone was defined by the presence of strong intraluminal echoes that were gravity-dependent or that attenuated ultrasound transmission (acoustic shadow).

We defined participants as having metabolic syndrome if they met at least three of the following criteria, as defined by the National Cholesterol Education Program (NCEP) Adult Treatment Panel III (ATP III) guidelines with adoption of the Asian criteria for abdominal obesity [23]: (1) hypertriglyceridemia with fasting plasma triglycerides ≧1.7 mmol/l; (2) low HDL-C with fasting HDL-C <1.03 mmol/l in men and <1.29 mmol/l in women; (3) high blood pressure with systolic or diastolic blood pressure ≧ 130/85 mmHg; (4) hyperglycemia with FPG ≧5.6 mmol/l; and (5) central obesity (WC>90 cm in men and >80 cm in women, based on the World Health Organization Asia-Pacific criteria).

All of the statistical analyses were performed using the 17^th^ version of the SPSS (Chicago, Illinois, USA) software. Continuous variables were expressed as the mean ± standard deviation, and categorical variables using numbers (percentages). Student's t test was used for the comparison of continuous variables, and categorical variables were compared between two groups using chi-square tests. In the multivariate analysis, the adjusted odds ratio (OR) and 95% confidence interval (CI) were calculated using a binary logistic regression model after adjustments for age, gender, obesity, current smoking, current alcohol drinking, regular exercise, education, hepatitis B, hepatitis C, and eGFR. Statistical significance was defined as a p value <0.05.

## Results

Of the 8,188 participants, 447 (5.5%) had gallstones, with 239 (5.1%) men and 208 (6.0%) women. Table 1 summarizes the clinical characteristics of study subjects with and without gallstones. Participants with gallstones were older and had a higher BMI, WC, SBP, DBP, FPG, total cholesterol, AST, ALT value, but a lower level of HDL-C than those without them. In addition, a low education level and hepatitis C were significantly more prevalent in subjects with gallstones, as compared to individuals without them.

**Table 1:** Clinical characteristics of the study subjects.

	Gallstones		
	Yes (n = 447)	No (n = 7,741)	P value
Age (yr)	52.1±12.5	45.5±11.8	<0.001
Gender, female	208 (46.5)	3254 (42.0)	0.061
Education (yr), 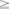 12	224 (53.7)	4781 (64.1)	<0.001
BMI (kg/m^2^)	24.3±3.3	23.8±3.4	0.002
WC (cm)	83.6±9.8	81.7±10.1	<0.001
SBP (mmHg)	126.5±18.1	123.5±18.0	<0.001
DBP (mmHg)	69.3±9.7	68.1±9.9	0.014
FPG (mmol/l)	4.93±0.51	4.87±0.48	0.021
Total cholesterol (mmol/l)	5.15±0.94	5.01±0.93	0.003
Triglycerides (mmol/l)	1.39±0.80	1.33±0.85	0.215
HDL-C (mmol/l)	1.26±0.336	1.30±0.35	0.042
eGFR (ml/min/1.73 m^2^)	93.2±16.9	97.2±17.5	<0.001
CKD	8 (1.8)	74 (1.0)	0.085
Hepatitis B	68 (15.2)	1063 (13.7)	0.348
Hepatitis C	28 (6.3)	219 (2.8)	<0.001
Current smoking	67 (17.3)	1245 (17.6)	0.877
Current alcohol drinking	59 (15.7)	1234 (17.7)	0.332
Regular exercise	235 (52.6)	3855 (49.8)	0.261
Components of metabolic syndrome			
FPG 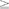 5.6 (mmol/l)	52 (11.6)	708 (9.1)	0.078
Blood pressure 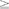 130/85 (mmHg)	175 (39.1)	2400 (31.0)	<0.001
Triglycerides 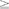 1.7 (mmol/l)	111 (24.8)	1743 (22.5)	0.255
HDL-C<1.03 mmol/l (M), <1.29 mmol/l (F)	174 (38.9)	2582 (33.4)	0.015
WC>90 cm (M), >80 cm (F)	171 (38.3)	2227 (30.1)	<0.001

Data are expressed as mean ± standard deviation (SD) or number (percentage).

BMI, body mass index; WC, waist circumference; SBP, systolic blood pressure; DBP, diastolic blood pressure; FPG, fasting plasma sugar; HDL-C, high density lipoprotein cholesterol; eGFR, estimated glomerular filtration rate; CKD, chronic kidney disease.

With regard to the components of metabolic syndrome, a significant difference existed in high blood pressure, low HDL-C, and central obesity between the subjects with and without gallstones. Among the subjects with different numbers of metabolic abnormalities, there was an increased prevalence of gallstones as the number of metabolic abnormalities increased (test for trend, p<0.001, table 2).

**Table 2:** The prevalence of gallstone in subjects with different numbers of metabolic abnormalities.

	Gallstones		Test for trend
	Number (yes/total)	Prevalence	P value
Number of metabolic abnormality			<0.001
None	105/2769	3.8	
One	132/2373	5.6	
Two	114/1617	7.1	
Three or more	96/1429	6.7	

The results of the multivariate analysis on the relationship between metabolic abnormalities and gallstones are summarized in Table 3. In Model 1, based on a reference group without metabolic abnormalities, those with one, two, and three or more metabolic abnormalities all had a greater risk of gallstones (age and gender-adjusted OR = 1.39, 1.67, and 1.56, respectively). After adjusting for age, gender, obesity, education level, and lifestyle factors, including current smoking, alcohol drinking, and regular exercise (Model 2), the relationship between metabolic abnormalities and gallstones remained significant (adjusted OR =  1.36, 1.41, and 1.57, respectively). In the final model (Model 3), we further adjusted for hepatitis B, hepatitis C, and CKD, and there was still a positive association between metabolic abnormalities and gallstones, with an increased odds ratio as the number of abnormalities increased (adjusted OR = 1.35, 1.40, and 1.59, respectively). In addition, hepatitis C (OR = 1.95, p = 0.006), but not hepatitis B or CKD, was positively associated with gallstones.

**Table 3:** The adjusted odds ratio and 95% confidence interval of the association between metabolic abnormalities and the risk of gallstones based on binary logistic regression.

	Model 1	Model 2	Model 3
Number of metabolic abnormalities			
1 vs 0	1.39 (1.06–1.81)*	1.36 (1.02–1.82)*	1.35 (1.01–1.80)*
2 vs 0	1.67 (1.26–2.21)**	1.41 (1.02–1.95)*	1.41 (1.01–1.94)*
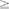 3 vs 0	1.56 (1.09–2.09)*	1.57 (1.11–2.24)*	1.59 (1.12–2.25)*
Age (yr)			
40–65 vs 20–40	2.05 (1.58–2.67)**	1.85 (1.40–2.45)**	1.83 (1.38–2.43)**
>65 vs 20–40	4.59 (3.22–6.53)**	3.82 (2.50–5.82)**	3.63 (2.36–5.57)**
Gender, female vs male	1.33 (1.09–1.61)*	1.21 (0.95–1.54)	1.21 (0.95–1.54)
BMI (kg/m^2^), 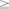 27 vs <27		0.86 (0.62–1.18)	0.85 (0.62–1.18)
Education (yr), 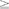 12 vs <12		0.92 (0.72–1.16)	0.94 (0.74–1.19)
Current smoking, yes vs no		1.13 (0.82–1.54)	1.13 (0.83–1.54)
Current alcohol drinking, yes vs no		0.93 (0.68–1.27)	0.94 (0.69–1.29)
Regular exercise, yes vs no		1.05 (0.85–1.30)	1.04 (0.84–1.29)
Hepatitis B, yes vs no			1.11 (0.82–1.51)
Hepatitis C, yes vs no			1.95 (1.21–3.15)*
CKD, yes vs no			1.32 (0.55–3.17)

* P<0.05, ** P<0.001.

BMI, body mass index; CKD, chronic kidney disease.

Model 1: Age, gender.

Model 2: Age, gender, obesity (BMI>27), current smoking, current alcohol drinking, regular exercise, education.

Model 3: Age, gender, obesity (BMI>27), current smoking, current alcohol drinking, regular exercise, education, hepatitis B, hepatitis C, CKD.

## Discussion

Although previous studies indicated that there is a strong association between metabolic syndrome and gallstones, few studies evaluated their association in a non-diabetic population [18], [19] without considering some important confounding factors, such as hepatitis C infection and CKD, simultaneously. The current study shows that non-diabetic subjects with metabolic syndrome and even one or two metabolic abnormalities had a higher risk of gallstones. This positive association remained even after adjusting for hepatitis C infection, CKD, and other important confounding factors. In addition, the risk of gallstones seems to be higher as the number of metabolic abnormalities increases. To the best of our knowledge, this is the first study to demonstrate the relationship between metabolic syndrome and gallstones in a non-diabetic population, independent of hepatitis C and CKD.

This study presented a positive association of metabolic abnormalities on gallstones in non-diabetic subjects who were neither under anti-hypertensive nor lipid-lowering treatment. If we included the subjects with treated hypertension and/or dyslipidemia, the positive association still existed in multivariate analysis model with an increased odds ratio as the number of abnormalities increased (OR = 1.39, 1.63, and 1.90, respectively). If MetS was used as a dichotomous variable in multivariate analysis, there was still a positive association between metabolic syndrome and gallstone, (OR = 1.38, p = 0.004), which is compatible with a previous study [7]. However, if we excluded the subjects with treated dyslipidemia or treated hypertension, the positive association did not reach the statistical cut-off of 0.05 (OR = 1.25, p = 0.099). The difference may be caused by subject selection. In our study, the study subjects were grossly healthy people. The result of our study emphasized the dose-dependent effect of the number of metabolic abnormality in a disease-free population, although did not show the significant difference between metabolic syndrome or not. In addition, there are several alternative definitions of MetS, such as NCEP-ATP III definition of MetS with the central obesity defined as greater than 40 inches (102 cm) in men or greater than 35 inches (88 cm) in women, and the International Diabetes Federation (IDF) consensus worldwide definition of MetS. The positive association of metabolic abnormalities on gallstones disappeared in the multivariate analysis when using definition of NCEP-ATP III (OR = 1.36, 1.31, and 1.54; p = 0.02, 1.11, and 0.03, respectively) or IDF (OR = 1.13, 1.27, and 1.26; p = 0.62, 0.21, and 0.18, respectively). However, when we included the patients with treated dyslipidemia and treated hypertension, the positive association became significant either under definition of NCEP-ATP III (OR = 1.38, 1.61, and 1.75; p = 0.01, 0.001, and <0.001, respectively) or IDF (OR = 1.14, 1.35, and 1.43; p = 0.57, 0.05, and 0.01, respectively).

Previous studies [7], [8] revealed that metabolic syndrome is strongly associated with an increased risk of gallstones, but their study subjects were not confined to non-diabetic ones. As for non-diabetic subjects, a positive association was found between metabolic syndrome and gallstones in a male population [18]. In addition, another study found that the positive association between metabolic syndrome and gallstones disappeared after adjustment for menopausal status in a female population [19]. One age- and gender- adjusted study [7] showed a dose-dependent effect of metabolic abnormalities on the risk of gallstones. In our non-diabetic subjects, as compared with the subjects without metabolic abnormalities, those with one, two, and three or more such abnormalities suffered from a 35, 40, and 59% higher risk of gallstones, respectively, after further adjustments for obesity, lifestyle, education, level, CKD, hepatitis B and C, in addition to age and gender. This means that the higher the number of metabolic abnormalities, the greater the risk of gallstones in non-diabetic subjects.

The exact pathogenic link between metabolic syndrome and gallstones remains unclear, but hyperinsulinemia may play a role in their relationship [9]. Ruhl and Everhart documented a positive association between hyperinsulinemia and gallstones in the large scale, carefully designed Third National Health and Nutrition Examination Survey [9]. Hyperinsulinemia may be an important factor that enhances gallstone formation by increasing the activity of hydroxyl-3-methylglutaryl-coenzyme [24], and directly stimulating the bile acid-independent flow of bile into perfused liver [25]. Hepatic insulin resistance also plays an important role in cholesterol gallstone formation by the following mechanisms: (1) disinhibition of the forkhead transcription factor FoxO1, via increasing expression of biliary cholesterol transporters Abcg5 and Abcg8, resulting in an increase in biliary cholesterol secretion, and (2) decreased expression of the bile acid synthetic enzymes, via partial resistance of FXR and its regulator, FGF-15, leading to a lithogenic bile salt profile [26]–[29]. In addition, recent studies showed that leptin [30] and some metabolic kinase signalings, such as LKB1-AMPK and -SIK3 [31]-[33] could regulate bile acid metabolism and might affect gallstone formation. However, more studies are needed to clarify the direct links between metabolic syndrome and gallstones.

In the current study, a positive association between hepatitis C infection and gallstones was observed in the multivariate analysis model. A prospective hospital-based study by Acalovschi et al. [13] showed a positive association between hepatitis C infection and gallstones, which is consistent with the finding in our study. Insulin resistance may be the link between metabolic syndrome, cholesterol gallstone disease, and hepatitis C infection [34]. Hepatitis C infection may also impair gallbladder mucosa function and enhance gallstone formation [35]. In addition, Lai et al. found that CKD was positively associated with gallstones (OR = 1.58, 95% CI = 1.01–2.47) in a hospital-based cross-sectional study [14], although the pathogenesis remains unclear. In our study, we also adjusted CKD in the final multivariate model, but the association between CKD and gallstones was not significant (OR = 1.32, 95% CI = 0.55–3.17).

There are several limitations in this study. First, it used a hospital-based cross-sectional design, and thus the results may not be able to be extrapolated to the general healthy population. Second, the subjects were all drawn from a Chinese population, and further study in other ethnicities is needed. Third, the serum insulin and leptin level, hepatic and peripheral insulin resistance were not measured in the current study. These markers were also deserved to be measured in clarifying the relationship between metabolic syndrome and gallstones in future study. Fourth, no detail information was available on family history of gallstones, oral contraceptive use, number of pregnancies, and menopausal status, all of which are known risk factors for gallstone formation.

In conclusion, our study shows that even in a non-diabetic population, independent of hepatitis C infection or CKD, a positive association still exists between metabolic syndrome and gallstones.
